# Descemet membrane endothelial keratoplasty combined with presbyopia‐correcting and toric intraocular lenses – a narrative review

**DOI:** 10.1186/s12886-023-03240-5

**Published:** 2023-11-25

**Authors:** Marina Rodríguez-Calvo-de-Mora, Carlos Rocha-de-Lossada, Vito Romano, Manuel Rodríguez-Vallejo, Joaquín Fernández

**Affiliations:** 1 Qvision, Department of Ophthalmology of VITHAS Almería Hospital, Almería, 04120 Spain; 2 Ophthalmology Department, VITHAS Málaga, Málaga, 29016 Spain; 3 Hospital Regional Universitario de Málaga, Plaza del Hospital Civil, S/N, Málaga, 29009 Spain; 4 Departamento de Cirugía, Área de Oftalmología, Universidad de Sevilla, Doctor Fedriani, S/N, Seville, 41009 Spain; 5 Ophthalmic Unit, Department of Medical and Surgical Specialties, Radiological Sciences, and Public Health, University of Brescia, Brescia, Italy; 6 Ophthalmic Unit, ASST Spedali Civili di Brescia, Brescia, Italy

**Keywords:** Endothelial keratoplasty, Descemet membrane endothelial keratoplasty, Multifocal intraocular lenses, Extended-depth of focus intraocular lenses, Toric intraocular lenses, Presbyopia‐correcting intraocular lenses

## Abstract

Fuchs endothelial corneal dystrophy (FECD) is the leading indication for EK and may coexist with cataract and presbyopia. Notably, the outcomes of phacoemulsification in FECD patients are not as favorable as those in eyes without this condition. Historically, only monofocal intraocular lenses (IOLs) were recommended for these patients. However, recent reports have described the implantation of Premium-IOLs (such as Multifocal IOLs, Enhanced Depth of Focus IOLs, and Toric IOLs) in FECD eyes undergoing cataract surgery and Descemet membrane endothelial keratoplasty (DMEK). While the results are encouraging, they are not as optimal as those from unoperated eyes, especially when comparing simultaneous procedures to sequential ones. It’s advised to perform the DMEK first to improve the accuracy of IOL calculations. Still, even successfully operated eyes may experience secondary graft failure or graft rejection after DMEK. The success rate of a secondary DMEK is typically lower than that of the initial procedure. Furthermore, if the postoperative thickness after DMEK is less than anticipated, laser enhancements might not be an option. There’s a pressing need for more controlled and randomized clinical trials to ascertain the safety and effectiveness of Premium-IOLs for FECD eyes. This narrative review aims to collate evidence on the use of Premium IOL technologies in eyes receiving EK and to underscore key points for surgeons performing EK combined with cataract surgery.

## Background

Endothelial keratoplasty (EK) techniques are currently the surgical procedures of choice for endothelial failure [1–4]. In EK procedures, the diseased Descemet membrane (DM) and endothelium complex are removed. Descemet stripping automated endothelial keratoplasty (DSAEK) involves the transplantation of a variable amount of posterior stroma plus DM and endothelium [5], whereas Descemet membrane endothelial keratoplasty (DMEK) involves the insertion of a thin graft composed only of DM and endothelium, achieving the restoration of the original anatomy of the cornea [6]. Of these techniques, DMEK is increasingly being performed because of its impressive results in terms of visual acuity [7], refractive stability, minimal postoperative hyperopic change [8], and low rates of complications [7, 9].

Fuchs endothelial corneal dystrophy (FECD) is currently the leading indication for EK, particularly DMEK [10–12]. Visual disturbances due to guttae typically affect middle-aged patients, predominantly women, who often present with concurrent opacities in the crystalline lens and presbyopia [13, 14]. Consequently, patients with FECD may require cataract surgery performed before [15, 16], after [17–19] or simultaneously (in a triple procedure) with EK [14, 20–23]. Traditionally, cataract surgery for patients with FECD involved the implantation of monofocal intraocular lenses (IOLs), as Premium IOLs were considered a relative contraindication [24]. However, recent scientific evidence suggests that Premium-IOLs-implantation may be safe and effective for selected patients with FECD undergoing cataract surgery, as long as biometry measurements and IOL implantation are performed after successful DMEK [16, 18, 19].

In recent years, multiple presbyopia-correcting IOLs have been introduced to the market. Multifocal IOLs (MIOLs) are frequently employed in cataract surgery because they provide favorable outcomes in terms of corrected and uncorrected distance (CDVA/UDVA), intermediate (UIVA/DCIVA), and near (UNVA/DCNVA) visual acuities, meeting patient needs and expectations [25]. However, patients may experience photic phenomena and a contrast sensitivity loss [24, 26]. On the other hand, extended depth of focus (EDoF) IOLs offer distance and intermediate visual acuities comparable to MIOLs; however, the near visual acuity is poorer [27]. EDoF IOLs have been proposed as an alternative in certain situations for patients who are not candidates for MIOLs, although evidence remains limited [28]. In addition to presbyopia, the correction of astigmatism with IOLs is of significant interest. Toric IOLs (TIOLs) have been proven to successfully address regular astigmatism, improving both postoperative visual acuity and patients’ vision-related quality of life [29]. These lenses have also been implanted in patients with previous, concomitant, or planned EK [14, 23, 30].

Considering the reported satisfactory and predictable outcomes of EK, especially DMEK, both patients’ and surgeons’ expectations continue to rise, transforming cataract and EK procedures into near-elective surgeries—particularly for FECD patients [14]. These individuals may not only desire improved distance visual acuity but also seek spectacle independence. The aims of this narrative review are to summarize the evidence regarding the use of Premium IOL technologies in eyes undergoing EK procedures and to emphasize the key points that surgeons should consider when conducting EK and cataract surgery.

## Main text

### Methods

A snowball search approach using PubMed was conducted to identify articles and case reports published until the 28th of February 2023 related to the use of premium IOLs in eyes undergoing EK, discarding all related to different topics. A review of all the identified abstracts published in English and Spanish was undertaken.

#### Cataract surgery and DMEK in Fuchs patients

FECD is a complex genetically autosomal dominant-inherited condition in which typically there is a trinucleotide repeat expansion in the transcription factor 4 (TCF4) gene [11, 31]. The slowly-progressive, characteristic guttae of FECD are excrescences of the thickened DM that cause depletion of corneal endothelial cells and subsequent corneal edema [11]. FECD is more common in older females [11, 12] from Anglo-Saxon [32, 33] Central, and Northern European patients [34–36]. FECD is currently the leading indication worldwide for corneal transplantation [10, 37–40].

Cataract surgery in FECD (either sequential or combined with DMEK) offers good outcomes, albeit inferior to those in regular eyes, with a similar safety profile [41, 42]. The outcomes of triple vs sequential DMEK in FECD have been discussed extensively, showing in general comparable visual, refractive and ECD outcomes and without major adverse events [20, 21, 42–44], albeit some authors report higher complications in triple DMEK [45, 46].

Endothelial cell density (ECD) decreases after cataract surgery in FECD, with reported losses one year after the phacoemulsification ranging from 8 to 10% [47, 48]. The recommended techniques to protect the endothelium when performing triple EK or cataract surgery in previous EK eyes are analogue to those used when performing cataract surgery in FECD eyes [48–53]. Regarding the IOL material, it is known that hydrophilic IOLs may develop calcification after EK due the use of intraocular gas tamponade, decreasing visual acuity and sometimes requiring an IOL exchange [54–56]. Consequently, hydrophobic IOLs should be used in EK eyes of in FECD eyes -in case a future EK might be necessary [55, 57].

Regarding the staging of the procedures, cataract surgery in FECD eyes has been performed before [15, 16], after [17–19] and at the same time (triple procedure) [14, 20–23, 30] as EK. Avoiding sequential surgeries in triple procedures has advantages from the economic, logistic and patients’ point of view [42, 44, 57]. The staging of cataract / EK surgery depends on one hand, on the severity of the corneal edema, the density of guttae, and/or the opacity of the crystalline lens; and on the other hand, on the age and refraction of the patient [58, 59]. Traditionally, a triple procedure was recommended in patients older than 50 years old [59] to avoid damaging the graft in the future cataract surgery [48] which frequently is performed earlier in these patients [59]. As a rule of thumb, a triple procedure was indicated in FECD eyes with significant cataract when ECD was less than 1.000 cells/mm^2^ or central corneal thickness (CCT) was higher than 640 microns [16, 57, 60]. However, CCT can greatly vary among individuals. Consequently, other tests have also been used to predict FECD eyes needing corneal transplantation: increased corneal backscatter measured by in vivo confocal microscopy [61], central cornea densitometry [62], evaluation of the subclinical edema [63, 64] and other topographic/tomographic features [15, 65, 66]. Guttae in FECD also cause glare and decreased contrast sensitivity [67]. Objective ways to evaluate the severity of guttae have been the automatic quantification [68] and the peripheral-to-central ratio of guttae [64].

The corneal and refractive changes that takes places in FECD eyes after DMEK have been previously described [8, 69]. Briefly: CCT in FECD is typically greater in the center compared to the periphery [70]. The progressive central bulging of the posterior cornea decreases the posterior corneal astigmatism [69] and increases the posterior radius of curvature (PRC) and posterior corneal asfericity [71, 72], causing oblate posterior curvatures due to stromal edema. In fact, the ratio of CCT compared to peripheral thickness at 4 mm has been proposed to grade FECD and assist in surgical decisions [69, 72]. The corneal de-swelling that occurs after successful DMEK surgery causes the posterior corneal surface to steepen, thereby achieving the normal eye state (from -5 to -6 D) [69], increasing posterior asphericity, and changing the total corneal refractive power (TCRP) from 1 to 1.38D [69, 73]. When comparing post-DMEK eyes with healthy corneas, PRC values were reported to be similar. However, there is no universal agreement regarding the postoperative change after DMEK of the anterior radius of curvature (ARC) and anterior surface elevation maps: some authors report that these values remained rather stable [14, 66, 73–76], whereas some authors found that ARC values were frequently flatter [66, 71, 74]. The changes in the anterior corneal surface after DMEK are critical for IOL calculation, which is typically conducted by estimating total corneal power from measurements of the anterior surface, assuming a constant relationship between PRC-ARC ratio in normal eyes [77].

The complexities regarding the changes in corneal shape and IOL calculations in FECD patients undergoing DMEK have been discussed extensively elsewhere [74, 76]. There is relative consensus that the changes in the corneal power after DMEK are associated with a hyperopic shift in refraction of about + 0.30 to + 0.50D compared to the preoperative period, which remains stable thereafter [8, 74, 77, 78]. However, larger amounts of hyperopic shifts in more decompensated corneas, and even myopic shifts, have also been described [8, 14, 73]. Several studies have reported the relationship between hyperopic shift with posterior oblate surfaces (positive Q values) [74, 77, 79], but some authors have not found any previous biometric tomographic value related to IOL calculation error [74].

Numerous approaches have been proposed for optimizing IOL power calculation in DMEK eyes [77, 80, 81]. Campbell et al. [77] described the use of modified corneal power based on preoperative Pentacam measurements, but this method failed to account for the possibility that the posterior cornea may change after surgery. The same problem was described using total keratometry from IOL Master 700 [80]. Diener’s approach [81], which estimates changes in the posterior cornea ratio, seems more suitable. Some surgeons simply aim for a myopic target of -0.75 to -1D with formulas developed for healthy eyes [14, 21, 75], taking into account the generally reported hyperopic shift ranges from + 0.5 to + 1D [14, 21, 81]. Some authors like Campbell et al. [77] find the Haigis formula to decrease the postoperative refractive shift after triple DMEK compared to HofferQ, SRK/T, Holladay I and Barrett. However, other authors like Knutsson et al. [76] did not find any differences in the prediction error between Haigis, Hoffer Q, Holladay SRK/T and new formulas like Kane and EVO 2.0. In more decompensated corneas, identified by higher CCT values (> 640/660 microns) and flatter, more oblate posterior curvatures, the myopic target may need to be increased to -1.25 to -1.75D [75, 82]. Other current approaches are to modify the anterior keratometry based on estimations of postoperative corneal changes [71] or alternatively optimizing the constant [76]. The refractive shift observed in the first eye after a triple procedure can be used to predict the extent of the change in the second eye [83].

IOL calculations for premium IOLs must be extremely accurate to prevent patient dissatisfaction due to postoperative residual refractive error [84]. Unfortunately, even if systematic shifts are avoided using the previously described approaches, if DMEK is conducted after or concurrently with cataract surgery, the percentage of eyes achieving ± 0.50 D is around 30–50% and those >  ± 1D range between 15–50% depending on the series [8, 14, 73, 74, 82]. This contrasts with the percentage of eyes achieving ± 0.50 D attainable in healthy eyes, which has been reported to be above 85% [85]. This lower accuracy may necessitate laser enhancement procedures, thus increasing the risk of complications due to the abnormally thin postoperative corneas that may occur after DMEK in some patients [15, 66]. Nevertheless, there is anecdotal evidence of good results of laser enhancements for residual errors after DMEK: Femto-LASIK has been performed in seven eyes by Fernández-Vega-Cueto et al. [86] and PRK in one eye by Moura-Coelho et al. [87]. However, if the postoperative CCT of the cornea is too thin after DMEK as described by Arnalich et al. (below 500 microns in 25% of eyes, with 50% of corneas decreasing below 500 microns in the thinnest point) [66, 86] or there is an alteration in corneal biomechanics as shown by Hernández et al. [88], caution is warranted if Premium IOLs are offered, as postoperative refractive procedures may not be feasible.

Challenging the traditional staging approach, Price et al. [18] performed first the DMEK in FECD eyes to improve the accuracy of the calculations for the secondary implant of presbyopia-correcting IOLs, with 75% of eyes achieving ± 0.50 D for cataract surgery after DMEK. In our opinion, this percentage may be further improved if the total corneal power is considered instead of just the anterior corneal surface, particularly when using a thick lens formula for intraocular lens power calculation after DMEK in stable corneas [89]. In order to implant Premium IOLs, the FECD should not be too advanced when performing DMEK, because stromal fibrosis may prevent optimal visual rehabilitation [90]. Once the corneal edema is completely resolved and the refraction and keratometric values are stable, phacoemulsification can be planned (Price et al. [18] waited from three to five months), provided that the surgery is performed with great care to prevent ECD loss [18, 48, 49] and/or to avoid TIOL rotation [23, 30]. Performing planned Premium IOL implantation after a successful DMEK may be supported by the work of other authors that only found a mild decrease of ECD in phacoemulsification in DMEK eyes (from 1535 ± 195 cells/mm^2^ to 1158 ± 250 cells/mm.^2^ at 6–12 months after cataract surgery) [17].

However, DMEK is an intraocular surgery that may entail potential complications, such as graft detachment, intraocular pressure rise, graft failure, graft rejection and microbial keratitis, among others [91]. Even if long term results after DMEK seem encouraging in terms of graft survival [92, 93], there may be a rejection (mean 1% over 2 years) [94] or a secondary graft failure of the DMEK graft over the course of years. Repeat DMEK visual results are known to be worse compared to primary DMEK [93], and the secondary DMEK may hinder the Premium IOL functioning. The advantage of performing DMEK before Premium IOL placement is that early DMEK complications, if any, have been already addressed.

In summary, when considering the implantation of a presbyopia-correcting intraocular lens in FECD eyes, surgeons should first evaluate the type of procedure they will perform: DMEK before, after, or combined with cataract surgery. If DMEK is conducted after or combined with cataract surgery, surgeons should not use the total corneal refractive power for IOL power calculation. Instead, the best clinically available options today are to modify the anterior keratometry based on estimations of postoperative corneal changes, adjusting the target, or alternatively optimizing the constant. However, even with these methods, we believe that the accuracy of IOL power calculation is not sufficiently precise to ensure a reasonable level of spectacle independence. On the other hand, consecutive cataract surgery in stable corneas after DMEK is a better option from the standpoint of IOL calculation, that should still be further studied. However, performing phacoemulsification after keratoplasty is not without risks, as the transplanted corneas may not show stability and may suffer complications over time. In any case, there is room for improvement in the calculation methods for IOL power, and these improvements could lead to increased confidence in selecting presbyopia-correcting IOL for FECD patients.

#### Presbyopia correcting lenses in Fuchs patients undergoing DMEK

Although MIOL implantation has been shown to be a safe and effective procedure for meeting patients’ visual acuity expectations at multiple distances and for reducing spectacle dependence [26], some adverse events may occur. These include loss of contrast sensitivity and an increased number of patients experiencing bothersome photic phenomena [26, 95]. These adverse events can be particularly problematic for patients with FECD, who may already experience visual disturbances, such as reduced visual acuity and contrast sensitivity, due to the presence of guttae [22, 67]. As a result, FECD has traditionally been considered a relative contraindication for MIOL implantation [16, 24]. In fact, in a recent Delphi consensus aimed at guiding general ophthalmologists, Romano et al. [96] considered endothelial dysfunction as an absolute contraindication for presbyopia-correcting IOLs implantation. However, Blau-Most et al. [97] suggested that presbyopia-correcting IOLs could be carefully considered for patients with grade 2–5 FECD. In a case series of 19 eyes with FECD, they compared the outcomes of implantation of 9 EDoF and 10 MIOLs to a control group, reporting slightly inferior results for FECD eyes. However, the conclusions of this study should be interpreted cautiously due to inconsistent outcomes. The authors reported that the mean UNVA achieved with EDoF IOLs was 0.04 logMAR, while the predicted error was 0.08 D. These outcomes, along with the UDVA of 0.17 logMAR, suggest a considerable myopic shift in these patients and indicate that an autorefractometer was used instead of subjective measurement of the eye’s refraction [98]. Moreover, the MIOL subgroup included both bifocal and trifocal MIOLs, making a correct interpretation of the outcomes challenging. These issues, coupled with the mixing of toric and spherical IOLs, introduce serious bias in interpreting the results. Perhaps the most interesting finding of the Blau-Most et al. [97] study is that 77.8% and 75% of the patients in the EDoF and MIOL subgroups, respectively, would opt for the same IOL implantation again. This percentage is not dramatically inferior to the control group, which reported 73.9% and 90.9%. Consequently, evidence supporting the use of presbyopia-correcting IOLs in Fuchs patients remains limited, with only one case series study suggesting that levels of satisfaction comparable to those in healthy eyes can be achieved.

Few clinical cases have been published for patients implanted with MIOLs and DMEK conducted either before (2 eyes) [18], after (9 eyes) [19] or sequentially (2 eyes) [16]. Price et al. [18] successfully performed a DMEK followed by MIOL implantation in order to improve accuracy of the IOL calculation in two FECD eyes (the others received EDoF IOLs, as described in the next section). Pereira et al. [19] performed DMEK in eyes (3 with FECD, 3 with pseudophakic bullous keratopathy, 2 following DM detachment, and 1 with toxic anterior segment syndrome) suffering from endothelial decompensation after MIOL implantation, achieving the following results: 6–8 months postoperative, CDVA was 20/30 Snellen (0.18 LogMar) or better in 4 eyes (two did not achieve this due to amblyopia and late-onset glaucoma, two required LASIK, and one did not achieve it due to residual astigmatism). In addition, other adverse events such as cystoid macular edema, re-bubble, and posterior capsular opacity were reported. Therefore, even though the authors conclude that DMEK can be an effective surgical procedure to treat endothelial decompensation after MIOL implantation without the need to remove the IOL, all these possible adverse events should be considered. However, of the cases where adverse events were resolved (6 eyes), they achieved Jaeger 2 (J2) in near vision. Finally, Nanavaty [16] performed sequential MIOL implantation and DMEK in both eyes of a myopic patient who desired spectacle independence but could no longer tolerate contact lenses and was not an ideal candidate for refractive laser vision correction. One year after the surgery, UDVA was 20/16 and UNVA of 20/20. Unfortunately, the authors did not describe how IOL power was calculated in these eyes with CCT > 640, which poses a high risk for a hyperopic shift as described in the previous section Table 1.

**Table 1:** DMEK and Premium IOLs

*Article*	*IOL type*	*Corneal illness*	*N. eyes*	*Staging of cataract/ DMEK*	*IOL*	*Visual outcomes*	*Refractive outcomes*	*% eyes within target*	*Complications*
*Pereira* [19]	*MIOL*	*3 PBK, 3 FECD, 2 DM detachment, 2 TASS*	*9*	*1º cataract, 2º DMEK*	*IOLs were previously implanted by other surgeons* *8* = *AcrySof® ReSTOR®* + *3.0 D* *1* = *AcrySof® ReSTOR®* + *3.0 D Toric T5 IOL*	*CDVA* > *20/20* = *25%* *UDVA* > *20/25* = *37.5%*	Refractive shift* *2* = + *0.625* *1* = *-0.25* *1* = *neutral* *Only possible to calculate in 4 eyes	*N/A*	*2 re-bubbling, 2 CME, 2 capsulotomy, 2 LASIK enhancement*
*Nanavaty* [16]	*MIOL*	*FECD*	*2*	*1º cataract, 2º DMEK*	*PanOptix*	*UDVA 20/16* *UNVA 20/20*	*N/A*	*N/A*	*No*
*Price* [18]	*EDoF (14) & MIOL (2)*	*FECD*	*16*	*1º DMEK, 2º cataract*	*Power calculation: PCI* + *US biometry* + *topography* *Formula: Holladay II (compared with Barrett Universal II and the Hill radial basis function)*	*Mean CDVA* = *20/20* *Mean UCDA* = *20/25*	*Mean SE* *0.05D (range -0.75 to* + *0.75D)*	*All eyes ended up 0.75 D of emmetropia*	*Capsulotomy, number not specified*
*Yokogawa* [23]	*TIOL*	*FECD*	*15*	*Triple*	*- Spherical power calculated with SRK/T with PCI* + *US biometry, then aimed spherical refractive target of -0.5/-1D* *- Toric calculation: Alcon online toric calculator. Pentacam values used for simulated front keratometry. Target postop. astigmatism: 0* *- IOL: AcrySof IQ toric*	*Mean CDVA* = *20/24* *UDVA* = *53.6%* > *20/25*	*Mean decrease in astigmatism 2.23* ± *1.10D to 0.87* ± *0.75D*	*Mean prediction error* = *0.77* ± *0.54 D (range 0.10–1.77 D* *WTR* = *1.01* ± *0.57 D (range 0.21–1.77)* *ATR* = *0.44* ± *0.30 D (range 0.10–0.79)*	*1 eye 43 degrees rotation*
*Trindade* [30]	*TIOL*	*FECD*	*4*	*Triple*	*PCI* *Barrett true-K formula* *IOL: SN6ATx*	*Median UDVA* = *LogMAR (0.12* ± *0.04) UDVA* = *100%* > *20/30*	*Refractive astigmatism (centroid) 0.09* ± *0.53D*	*N/A*	*1 re-bubbling*
*Schoenberg* [14]	*TIOL*	*FECD*	*8*	*Triple*	*- Spherical power calculated with SRK/T with PCI* + *US biometry, then aimed for a spherical refractive target individualized to patient´s preferences plus -0.50D* *- Toric calculation: Alcon online toric calculator. Pentacam values used for simulated front keratometry. Target postop. astigmatism: 0* *- IOL: AcrySof IQ toric*	*Mean CDVA* = *20/20* *Mean UDVA* = *20/30*	*Refractive astigmatism 0.94* ± *0.90D*	*N/A*	*1 eye no improvement in refractive astigmatism*

*IOL* Intraocular Lens, *MIOL* Multifocal Intraocular Lens, *EDoF* Extended-depth of Focus Intraocular Lens, *TIOL* Toric Intraocular Lens, *PBK* Pseudophakic Bullous Keratopathy, *FECD* Fuchs Endothelial Corneal Dystrophy, *DM* Descemet Membrane, *TASS* Toxic Anterior Segment Syndrome, *DMEK* Descemet´s Membrane Endothelial Keratoplasty, *PCI* Partial coherence interferometry, *CDVA* Corrected-distance visual acuity, *UDVA* Uncorrected-distance visual acuity, *UNVA* Uncorrected-near visual acuity, *SE* Spherical Equivalent, *WTR astigmatism* With-the-rule-astigmatism

Regarding other presbyopia-correcting IOLs, such as EDoF lenses, the previous study by Price et al. [18] evaluated the visual outcomes of 14 eyes implanted with a diffractive EDoF IOL after prior DMEK (2 toric). The mean postoperative UDVA and UNVA were 0.08 ± 0.09 and 0.17 ± 0.14 logMAR, respectively, with all eyes having an SE ranging from -0.88 to + 0.75 D (71.43% within ± 0.5 D, mean -0.02 ± 0.53 D, and mean absolute 0.43 ± 0.3 D). These outcomes are comparable to those typically reported with EDoF IOLs and may even be slightly better [99]. However, the testing methods for measuring UNVA and distance were not reported by the authors. This figure compares favorably with reports from other authors who combined DMEK and cataract surgery, in which only 50 to 70% of eyes were within 1D of emmetropia.

In conclusion, only three studies have reported the outcomes of presbyopia-correcting IOL implantation in combination with DMEK. Although all authors concluded that presbyopia-correcting IOLs are an acceptable alternative for this population, the outcomes remain disputed. Pereira et al. [19] reported a notable number of adverse events in a small sample study, in contrast to Price et al. [18] reported no adverse events in a similarly constrained study. Additionally, although the most favorable outcomes were documented by Price et al. [18] using an EDoF IOL, the two eyes incorporated with MIOLs also demonstrated outcomes analogous to those in a healthy population. As a result, the evidence remains insufficient to conclusively recommend presbyopia-correcting IOLs, particularly MIOLs, for use in FECD-affected eyes.

#### Toric intraocular lenses and Fuchs patients undergoing DMEK

TIOLs correct regular astigmatism and improve postoperative visual acuity, enhancing patients’ vision-related quality of life [29]. Corneal astigmatism is not significantly altered after EK [8, 22] except for patients with remarkable differences in total astigmatism in the central 3.0 and 5.00 mm, where it can decrease postoperatively [100].

TIOLs have been implanted in FECD patients undergoing DSAEK [22] and DMEK, utilizing different staging strategies [14, 23, 30]. Yokogawa et al. [23] executed a triple DMEK, implanting TIOL (Acrysof Toric) in 15 FECD eyes and yielding good postoperative outcomes: 61.5% achieved an UDVA > 20/40 and 53.6% were > 20/25. The mean residual cylinder was 0.87 ± 0.75D. However, these results are inferior to TIOL implantation in regular cataract surgery, where > 91% of eyes achieved an UDVA > 20/40 and > 60% were > 20/25, with postoperative refractive astigmatism of 0.59 ± 0.72D. An overcorrection was observed in those eyes with with-the-rule astigmatism (WTR), likely due to the posterior against-the-rule (ATR) corneal astigmatism in FECD patients [23]. Posterior ATR has been described in the normal population [101–103] and after DMEK [104]. By combining TIOL calculators and biometric measurements [105], obtaining epithelial maps through anterior segment optical coherence tomography [106, 107] and performing anastigmatic temporal incisions [108–110] may improve the accuracy of TIOL calculations. Yokogawa et al. [23] hypothesize that the maneuvers performed during the insertion and manipulation of the DMEK graft, in conjunction with the air-bubble, may cause a clockwise rotation of the TIOL. To circumvent this issue, the authors recommend re-checking the alignment of the TIOL once the DMEK graft is fixated and the air bubble is in place. Trindade et al. [30] also conducted a triple DMEK implanting a TIOL (Acrysof Toric, SN6ATx Alcon) in four non-decompensated FECD eyes. Median UDVA was LogMAR 0.12 ± 0.04, all eyes achieved UDVA > 20/30 (0.18 LogMar) and the centroid at the corneal plane of refractive astigmatism was 0.09@125º ± 0.53D. The authors assert that performing triple DMEK with TIOL in eyes before clinical edema develops enables the TIOL calculations to be more accurate [30]. Schoenberg et al. [14] reported the outcomes of eight eyes undergoing triple DMEK with TIOLs from a larger series. Median postoperative UDVA was 20/30 (range 20/25–20/50), while median CDVA values achieved were 20/20 (range 20/15–20/30). No significant differences were found in UDVA (*p* = 0.71), nor in CDVA (*p* = 0.25), compared to the eyes with regular IOL implantation. Refractive astigmatism after the Triple DMEK with TIOL implantation was + 0.94 ± 0.90 D. One eye did not show any improvement in postoperative astigmatism despite proper alignment of the TIOL Table 1.

In summary, corneal astigmatism seems to remain stable after DMEK. TIOLs have been used in FECD eyes with good results. Proposed strategies to increase the accuracy of TIOL calculations include: combining TIOL calculators and biometric measurements [105], obtaining epithelial maps through anterior segment optical coherence tomography [106, 107] and performing anastigmatic temporal incisions [108–110]. Rechecking the alignment of the TIOL once the DMEK graft is fixed may prevent TIOL rotation [23] and performing triple DMEK with TIOL in eyes before clinical edema develops may enable the TIOL calculations to be more accurate [30].

## Conclusions

Premium IOLs have been traditionally indicated only in healthy eyes. Recent scientific evidence suggests that presbyopia-correcting IOL and TIOL implantation may be safe and effective for selected patients with FECD undergoing cataract surgery and DMEK, but their results may be inferior compared to unoperated eyes if a triple vs a staged procedure is performed. Staging first the DMEK procedure and performing the cataract surgery in a second time can improve the accuracy of IOL calculations, but there is a potential risk of significant ECD loss. Moreover, the decreased pachymetry found in some post-DMEK corneas may preclude the performance of postoperative laser enhancements. Finally, if a secondary DMEK is needed over time, the functioning of the Premium IOLs may be hindered. More research, especially in form of controlled and randomized clinical trials, are needed to confirm the safety and efficacy of presbyopia-correcting IOLs and TIOL in FECD eyes.

## Data Availability

None.

## Abbreviations

ARC: Anterior Radius of Curvature
ATR: Against-The-Rule Astigmatism
CCT: Central Corneal Thickness
CDVA: Corrected Distance Visual Acuity
DCIVA: Distance-Corrected Intermediate Visual Acuity
DCNVA: Distance-Corrected Near Visual Acuity
DM: Descemet Membrane
DMEK: Descemet Membrane Endothelial Keratoplasty
DSAEK: Descemet Stripping Automated Endothelial Keratoplasty
ECD: Endothelial Cell Density
EDoF: Extended Depth of Focus
EK: Endothelial Keratoplasty
FECD: Fuchs Endothelial Corneal Dystrophy
IOLs: Intraocular Lenses
LogMAR: Logarithm of the Minimum Angle of Resolution
MIOLs: Multifocal Intraocular Lenses
PRC: Posterior Radius of Curvature
TCRP: Total Corneal Refractive Power
TIOLs: Toric Intraocular Lenses
UDVA: Uncorrected Distance Visual Acuity
UIVA: Uncorrected Intermediate Visual Acuity
UNVA: Uncorrected Near Visual Acuity
WTR: With-The-Rule Astigmatism
